# Longitudinal association of exposure to work-related stress with major depressive disorder and the role of occupational burnout in this association in the general population

**DOI:** 10.1007/s00127-024-02735-w

**Published:** 2024-08-31

**Authors:** Yara Shoman, Setareh Ranjbar, Marie-Pierre F. Strippoli, Roland von Känel, Martin Preisig, Irina Guseva Canu

**Affiliations:** 1 Department of Occupational and Environmental Health, Center of Primary Care and Public Health (Unisante), University of Lausanne, Lausanne, Switzerland; 2 Department of Psychiatry, Psychiatric Epidemiology and Psychopathology Research Center, Lausanne University Hospital and University of Lausanne, Prilly, Switzerland; 3 Department of Consultation-Liaison Psychiatry and Psychosomatic Medicine, University Hospital Zurich, University of Zurich, Haldenbachstrasse 16/18, 8091 Zurich, Switzerland

**Keywords:** Major depressive disorder, Diagnosis, Work-related stress, Burnout, Esteem

## Abstract

**Purpose:**

To prospectively assess (1) the associations of Effort-Reward Imbalance (ERI), its individual components, and over-commitment with (a) the onset of a Major Depressive Episode (MDE) during a 3.6-year follow-up in a population-based cohort in participants with no current Major Depressive Disorder (MDD) in the beginning of the follow-up (*n* = 959), (b) incidence of MDD in the subsample of participants exempt from lifetime MDD (*n* = 490), and (c) the onset of a new MDE (i.e. recurrence) in the subsample of participants with remitted but no current MDD (*n* = 485), and (2) potential effect modification of burnout on these associations.

**Methods:**

DSM-IV Axis-I disorders were elicited using the semi-structured Diagnostic Instrument for Genetic Studies at each investigation. The ERI Questionnaire was used to measure ERI and overcommitment. Burnout was measured with the Maslach Burnout Inventory General Survey. Serially adjusted logistic regression models were used. The effect of burnout dimensions on these associations was assessed by testing interactions between the ERI and burnout dimensions.

**Results:**

(1) ERI was prospectively associated with the onset of MDE, even after adjustment for burnout [OR (95CI) = 1.22 (1.003–1.49)]. (2) The association between ERI and MDD incidence became non-significant after adjusting for burnout. (3) ERI was not associated with recurrence of pre-existing MDD. (4) burnout did not interact with ERI.

**Conclusions:**

Our results support a longitudinal association between ERI and the risk of onset of MDE in the community. Burnout did not modify this effect, but it may partially account for the association between ERI and MDD incidence.

**Supplementary Information:**

The online version contains supplementary material available at 10.1007/s00127-024-02735-w.

## Introduction

Major depressive disorder (MDD) is defined by the occurrence of one or multiple depressive episodes throughout a person’s life [3]. These depressive episodes can last for varying periods, ranging from months to years. Following such episodes, most patients typically return to their usual state of health. While some individuals may experience only one episode in their lifetime, the majority will face two or more. The prevalence of MDD is increasing worldwide, especially after the pandemic of COVID-19 [49], a rising economic burden globally. According to the Global Burden of Diseases in 2019, the rate of MDD prevalence was 1.8 and 3.0% in males and females, respectively [66]. Furthermore, depressive disorders were ranked the thirteenth leading cause of disability-adjusted life years (DALYs) worldwide in 2019 [18]. MDD is associated with higher direct and especially indirect costs in all age groups compared to non-depressed people [29]. MDD also leads to economic loss in the workplace due to lower productivity, increased presentism, absenteeism, early retirement, and unemployment across various countries [7, 17, 25, 28]. Consequently, MDD is a major occupational and global public health concern [35, 71].

The etiology of MDD has been debated [20], although it is generally believed to be caused by multiple factors [4]. These factors can be categorized into four primary dimensions: sociodemographic, physical illness, genetic/hereditary factors, and adverse childhood experiences (ACEs) [12]. Occupational factors were identified as risk factors for common mental health outcomes, especially depression [40]. Work stress is among the classical or well-known occupational factors and its role as a risk factor for depression has been shown [67]. Work stress can be defined as the harmful response of the worker’s inability to adapt to job demands at the workplace [27]. There are several models and indicators of work-related stress in the literature [2], such as the job demands resources model [15], and the Effort-Reward Imbalance (ERI) and overcommitment model [57]. Some work-related factors have been identified as contributors to depression based on findings from a meta-analysis [63]. Another meta-analysis of the associations between job strain and MDD concluded that job strain may expedite the development of clinical depression [34]. However, the results of a more recent meta-analysis showed that after considering statistical uncertainties as well as the potential for bias and confounding, it remains unclear which psychosocial workplace exposures are probable or improbable causes of depressive episodes or recurrent depressive disorders [38]. Moreover, it remains unclear which work-related factors predict depression based on the findings of the three previous meta-analyses since some of the occupational factors have been considered more in the literature than others [46].

The association between ERI and depression was assessed in three meta-analyses [38, 50, 59]. The results of two meta-analyses showed that workers exposed to ERI had an elevated risk of depression [50, 59]. However, the meta-analysis of Mikkelsen et al. 2021 [38] revealed low consistency of risk estimates. One reason for the large variance in results was the use of different diagnostic methods to assess depression across studies. Indeed, the 51 studies included in this meta-analysis measured depression using “semi-structured interviews, fully structured interviews, self-administered questionnaire instruments, questionnaire self-reported doctor’s diagnosis, and register information on antidepressant treatment or hospital discharge diagnoses”. Only three studies relied on semi-structured diagnostic interviews, the gold standard to assess depression, which minimizes the risk of misclassification. Also, Rugulies et al, 2017 [50] and Siegrist et al. 2020 came to similar conclusions suggesting that future studies should assess depression using diagnostic interviews. Among the three studies using diagnostic interviews to assess depression, only one study evaluated the association between ERI and depression [68] whereas the other two studies assessed the association between other work-related factors and depression. Therefore, we are aware of only one study that assessed the association between ERI and incidence of depression in which depression was measured using a structured interview [68]. Moreover, we are not aware of any study on the association between ERI and the risk of recurrence in pre-existing depressive disorders.

One example of low control of confounding factors is that the role of burnout in the association between ERI and depression has not previously been investigated. This is important because burnout was reported as a mediator in the association between job demands and psychosomatic health complaints (Schaufeli and Bakker, 2004) and in the association between work-related quality of life and depressive symptoms [45].

Other limitations of existing studies are the measurement of ERI using self-reports, a low level of confounder control, and publication bias [38]. Potential confounding variables should be considered based on the literature: demographic and lifestyle factors (age, sex, physical activity, and smoking status) [50], anxiety disorders [24, 37], illicit drug use disorder [13], and alcohol use disorder [9].

An unresolved question is the role of burnout in the association between ERI and the onset of depression. One previous study found burnout to be a mediator in the association between work-related quality of life and depressive symptoms [45]. However, following a population-based cohort over more than 3 years, we did not find a prospective association between ERI and burnout (Shoman 2023), which makes it unlikely that burnout could mediate the association between ERI and the onset of depressive episodes. For the present paper, we used the same cohort [19, 48], which presents an ideal setting to overcome the limitations of previous studies. It allows investigating the association between exposure to work-related stress, defined by the ERI and overcommitment model, and MDD using clinical diagnostic interviews for the diagnosis of MDD taking into consideration multiple potential confounders. Although we have already shown the absence of a prospective association between ERI and burnout, which makes it unlikely that burnout mediates the potential prospective association between ERI and MDD, we cannot preclude that burnout could confound or modify this association.

Therefore, using data from this prospective population-based cohort, the present study aimed to prospectively assess (1) the associations of ERI, its individual components (effort, reward), and over-commitment with (a) the onset of a Major Depressive Episode (MDE) during a 3.6-year follow-up in participants with no current MDD in the beginning of the follow-up (whole sample), (b) the incidence of MDD in the subsample of participants exempt from lifetime MDD in the beginning of the follow-up, and (c) the onset of a new MDE (i.e. recurrence of MDD) in the subsample of participants with remitted but not current MDD in the beginning of the follow-up; and (2) potential effect modification (interaction) of occupational burnout measured in the beginning of the follow-up on these three longitudinal associations.

## Methods

### Study sample and follow-up

**Fig. 1:**
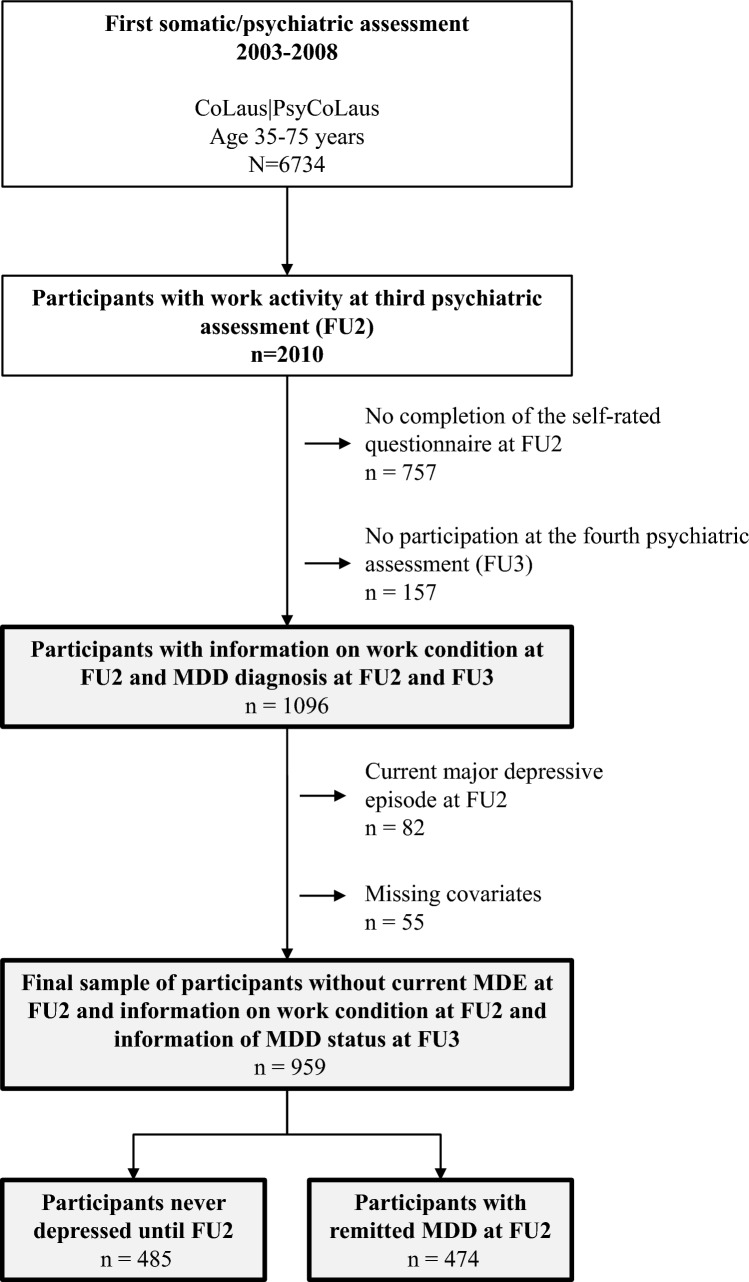
Flowchart for the selection of participants. *FU* follow-up, *MDD* major depressive disorder, *MDE* major depressive episode

The present data stem from the prospective cohort study CoLaus|PsyCoLaus, designed to assess cardiovascular risk factors and mental disorders in the community as well as their associations. The methodological features of the recruitment and baseline assessments of CoLaus|PsyCoLaus have been described in detail elsewhere [19, 48]. Briefly, CoLaus|PsyCoLaus includes a random sample of 6734 participants (age range: 35–75 years) selected from the general population according to the civil register of the city of Lausanne (Switzerland). After a first physical and psychiatric investigation, which took place between 2003 and 2008, the cohort was followed up after approximately five (first follow-up, FU1), nine (second follow-up, FU2), and 13 years (third follow-up, FU3). At FU2, 2010 participants with professional activity or professional inactivity of less than one year were invited to fill in self-reported questionnaires on ERI, over-commitment, and burnout during the psychiatric evaluation (Fig. 1). Among them, 1096 completed them and participated in the psychiatric evaluation at FU3. A total of 82 participants were excluded because they reported current MDE at FU2 and 55 because of incomplete data on potential confounders, resulting in a final sample of 959 participants. Among them, 485 participants had no lifetime history of MDD at FU2 and 474 participants reported remitted MDD at this assessment.

### Measurement of study variables

#### Assessment of psychiatric disorders

**Fig. 2:**
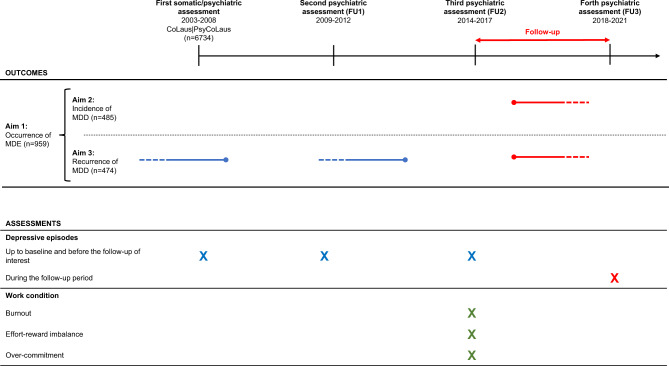
Data assessments and definitions of the three outcomes. *FU* follow-up, *MDD* major depressive disorder, *MDE* major depressive episode.  remitted MDD.  MDE during the follow-up

Diagnostic information on mental disorders including lifetime MDD was elicited at each psychiatric evaluation using the French version [30] of the semi-structured Diagnostic Interview for Genetic Studies (DIGS) [44]. At the first psychiatric evaluation the DIGS assessed psychiatric disorders across lifetime, whereas the DIGS version used at each follow-up assessed psychiatric disorders that occurred during the previous follow-up period (Fig. 2). The inter-rater and test–retest reliability of the French version of this DIGS is considered adequate for major mood [47] and substance use disorders [6]. The DIGS was completed with anxiety disorders sections of the French version of the Schedule for Affective Disorders and Schizophrenia-Lifetime and Anxiety disorder version (SADS-LA) [16, 31]. Lifetime diagnoses at the ERI assessment (FU2) and diagnoses reporting during the follow-up were assigned according to the Diagnostic and Statistical Manual of Mental Disorders (DSM-IV) (American Psychologists Association, 1994). Lifetime diagnoses until the ERI assessment (FU2) were subdivided into three categories: current (i.e., meeting DSM-IV criteria) at the time of the ERI assessment, remitted (i.e., not meeting criteria at the time of the ERI assessment, but having previously met these criteria) and never (i.e., having never met lifetime criteria up to the ERI assessment). MDD status during the follow-up was defined as the occurrence of a MDE during the interval. Additionally, the DIGS collects information on smoking habits and physical activity with a minimum frequency of once a week. Interviewers were master-level graduate psychologists, who were trained over a one- to two-month period. Each interview and diagnostic assessment were scrutinized by an experienced psychologist.

#### Effort-reward imbalance, over-commitment, and burnout

ERI and over-commitment were assessed using the French-validated version [41] of the effort-reward imbalance Questionnaire [57]. This questionnaire consists of 23 items split into three subscales: effort, reward (i.e., esteem, promotion, and security), and over-commitment. The items are scored on a 5-point Likert scale for effort and reward and on a 4-point Likert scale (i.e., from 1 “Strongly disagree” to 4 “Strongly agree”) for over-commitment. Examples of items from the esteem dimension are “I receive the respect I deserve from my supervisors”, and “I receive the respect I deserve from my colleagues”. We calculated a ratio for the imbalance between effort and reward: effort/reward*correction factor with the correction factor of 0.55 for balancing the unequal number of items of the dimensions of effort and reward subscales.

Burnout was assessed using the French-validated version [8] of the 16-item Maslach Burnout Inventory-General Survey (MBI-GS) [53]. The items are scored on a 7-point Likert-type scale from 0 “never” to 6 “every day”. There are three dimensions of the MBI-GS: exhaustion, cynicism (negatively worded), and professional efficacy (positively worded).

Participants received the self-administered questionnaire during the psychiatric assessment.

### Statistical analysis

First, descriptive statistics using percentages (for categorical variables) and means, and standard deviations (SD) or median and interquartile range (IQR) (for the continuous variables) were presented and Chi-Square tests for categorical variables and t-test or Wilcoxon tests (when the variable is not normally distributed) for continuous variables were used to compare the participants according to the MDD status during the four-year follow-up. We scaled all continuous variables by transforming them to z-scores before using them in the regression. Two separate sets of serially adjusted logistic regression models were fitted: one set of models included the effort-reward ratio and the over-commitment dimensions as independent variables; the other set of models included the ERI dimensions [effort, the three reward sub-dimensions (esteem, promotion, and security)] and over-commitment as independent variables. For the first study goal, the dependent variable was the onset of an MDE during follow-up. The first model (Model 1) was adjusted for remitted MDD status at the first assessment. Model 2 was additionally adjusted for age, sex, and the duration of follow-up. Model 3 was further adjusted for additional potential confounding variables at the first assessment (physical activity, smoking status, psychiatric disorders [anxiety disorders (generalized anxiety disorder, panic disorder, agoraphobia, social phobia), illicit drug and alcohol use disorders (abuse or dependence)]. Finally, Model 4 was further adjusted for the three burnout dimensions (exhaustion, cynicism, and professional efficacy).

For study goal two, Models 1, 2, 3, and 4 were conducted in the subsample of participants exempt from a lifetime history of MDD at the first assessment. Hence, the onset of an MDE during follow-up corresponded to the incidence of MDD during this period. For study goal three, Models 1, 2, 3, and 4 were conducted in the subsample of participants with remitted MDD at the first assessment. Hence, the onset of an MDE during follow-up corresponded to the recurrence of MDD during this period.

For the fourth study goal (assessment of the effect of burnout dimensions at the first assessment on the associations between ERI at the first assessment and the onset of an MDE during follow-up), we tested interactions between ERI ratio and burnout dimensions in the previously described Models 4 for all three previously mentioned Aims 1, 2 and 3. All statistical analyses were performed using Stata statistical software version 16 [62] and a *p*-value < 0.05 was considered statistically significant. We checked the multicollinearity between variables using the correlation matrix, and none of the independent variables included in the models have correlation coefficients higher than 0.64 in absolute value (Supplementary Table S1, S2, and S3).

## Results

### Characteristics of the participants

Among the 959 participants without current MDE at the first assessment, half (50.6%) reported previously MDD. Seventeen percent of the participants reported MDE during the four-year follow-up. The mean (SD) age of the participants was 56.3 (6.2) years, and 49% were men (Table 1). A total of 41% were non-smokers, 80% were physically active, 9% reported illicit drug use disorder (abuse/dependence), 13% alcohol use disorder (abuse/dependence), and 22% any anxiety disorders. Participants with MDE during the four-year follow-up were more female, reported more frequently anxiety disorders, a higher score of exhaustion, cynicism, effort-reward ratio, effort, and over-commitment, and a lower score of security, esteem, and promotion than those without MDE during the four-year follow-up (all *p* < 0.01, Table S4). Among the 485 participants free from a lifetime history of MDD until the first assessment, 7% reported MDD during the four-year follow-up. Characteristics of these participants are presented in Table 1. Among participants who were never depressed before the first assessment, those with MDD during the four-year follow-up reported more frequently anxiety disorders, a higher score in exhaustion, cynicism, and effort-reward ratio but a lower score in esteem compared to those not reporting MDD during the four-year follow-up (Table S5). Among the 474 participants with remitted MDD at the first assessment, 27% reported an MDE during the four-year follow-up. Characteristics of these participants are also presented in Table 1. Among participants with remitted MDD at the first assessment, those reporting MDD during the four-year follow-up were mostly women and reported more frequently anxiety disorders, a higher score in exhaustion, cynicism, effort-reward ratio, and over-commitment but a lower score in security and promotion compared to those not reporting MDD during the four-year follow-up (Table S6).

**Table 1:** Description of the total sample and the subsamples by lifetime MDD status in the beginning of the follow-up

	Total sample (*N* = 959)	Lifetime MDD status in the beginning of the follow-up	
		No MDD (*N* = 485)	Remitted MDD (*N* = 474)
	Mean (SD) or percentage	Mean (SD) or percentage	Mean (SD) or percentage
Demographic characteristics			
Age in the beginning of the follow-up (years), mean (SD)	56.34 (6.17)	56.94 (6.44)	55.73 (5.83)
Male sex, %	49.3	62.1	36.3
Length of follow-up (years), mean (SD)	3.62 (0.54)	3.64 (0.53)	3.59 (0.54)
Behavioral characteristics in the beginning of the follow-up			
Smoking status, %			
Active smokers	9.4	9.9	8.8
Former smokers	49.4	46	53
Non-smokers	41.2	44.1	38.2
Physical activity^a^, %	79.9	81	78.7
Lifetime psychiatric disorder in the beginning of the follow-up			
Illicit drug use disorder (abuse/dependence), %			
Current	0.3	0.4	0.2
Remitted	8.1	6.8	9.5
Never	91.6	92.8	90.3
Alcohol use disorder (abuse/dependence), %			
Current	2.2	2.9	1.5
Remitted	10.9	10.9	11
Never	86.9	86.2	87.6
Anxiety disorders^b^, %			
Current	2.1	1.2	3
Remitted	19.7	14.2	25.3
Never	78.2	84.5	71.7
Burnout scores^c^ in the beginning of the follow-up, mean (SD)			
Exhaustion	1.44 (1.17)	1.17 (1.06)	1.71 (1.22)
Cynicism	1.67 (1.34)	1.50 (1.27)	1.85 (1.39)
Professional efficacy	4.63 (0.89)	4.66 (0.94)	4.60 (0.85)
Effort-reward imbalance scores in the beginning of the follow-up, mean (SD)			
Ratio	0.26 (0.13)	0.24 (0.12)	0.27 (0.14)
Effort	12.19 (3.92)	11.72 (3.69)	12.67 (4.10)
Reward			
Security	6.98 (1.80)	7.08 (1.69)	6.88 (1.91)
Esteem	11.63 (1.99)	11.70 (1.67)	11.55 (2.27)
Promotion	8.48 (1.67)	8.59 (1.56)	8.36 (1.78)
Over-commitment	13.82 (3.64)	13.57 (3.55)	14.09 (3.72)

*MDD* major depressive disorder, *MDE* major depressive episode, *SD* standard deviation

^a^At least once a week

^b^Generalized anxiety disorder, panic disorder, agoraphobia or social phobia

^**c**^Measured using Maslach Burnout Inventory (MBI)

### Association between ERI, over-commitment, and MDD during a four-year follow-up

Associations between ERI and over-commitment and the onset of MDE during a four-year follow-up are presented in Table 2. Among participants not currently depressed at the first assessment, only a higher effort-reward ratio (imbalance) was associated with a higher likelihood of MDE onset during the four-year follow-up when adjusting for potential confounders (Table 2, Models 1, 2, and 3). This association remained statistically significant with a slight decrease in the effect size after adjustment for the three burnout dimensions (Table 2, Model 4). The effect sizes of the associations between over-commitment and MDE onset were higher (*p* < 0.05 for the chi-squared test of the difference between the effect sizes of the two variables in the regression) compared to the effect sizes of the associations between other ERI dimensions and MDE onset. However, the association between over-commitment and MDE onset was not statistically significant. Although the pseudo-R squared slightly improved from Model 1 to Model 4, the Akaike Information Criterion (AIC) for Model 2 was the lowest indicating that Model 2 performance was the best. Among participants free from a lifetime history of MDD until the first assessment, a higher effort-reward ratio (imbalance) was associated with an increased likelihood of MDD incidence during the four-year follow-up, (Table 3, Models 1, 2, and 3) but this association became not significant after adjusting for the three burnout dimensions (Table 3, Model 4). Although the pseudo-R squared slightly improved from Model 1 to Model 4, the AIC for Model 1 was the lowest. Among the reward dimensions, a lower score in esteem was associated with a lower likelihood of MDD incidence during the four-year follow-up even after full adjustment (Table 3). Among participants with remitted MDD at the first assessment, neither the ERI ratio, ERI dimensions nor over-commitment were associated with the recurrence of MDD before or after adjustment for the burnout dimensions (Table 4). The pseudo-R squared slightly improved from Model 1 to Model 4, but the AIC for Model 2 was the lowest indicating that Model 2 performance was the best.

**Table 2:** Results of logistic regression models of the associations of work stress modeled as Effort-Reward Imbalance (ERI) or its dimensions, and over-commitment in the beginning of the follow-up with the onset of a Major Depressive Episode during the follow-up in the whole sample (*n* = 959)

Predictors	Model^a^ Odds ratio (95%CI)	Model^b^ Odds ratio (95%CI)	Model^c^ Odds ratio (95%CI)	Model^d^ Odds ratio (95%CI)
Effort-reward ratio	1.31** (1.10–1.57)	1.32** (1.11–1.58)	1.32** (1.10–1.58)	1.22* (1.003–1.49)
Over-commitment	1.16 (0.94, 1.44)	1.17 (0.95, 1.45)	1.14 (0.92, 1.42)	1.12 (0.89, 1.42)
Pseudo *R*^2^	0.07	0.09	0.10	0.11
AIC	733.3009	727.6759	735.7162	737.4839
Effort	1.10 (0.88–1.38)	1.11 (0.88–1.40)	1.12 (0.89–1.41)	1.05 (0.82–1.35)
Security	0.87 (0.72–1.04)	0.85 (0.71–1.02)	0.87 (0.72–1.04)	0.89 (0.74–1.07)
Esteem	0.88 (0.73–1.06)	0.89 (0.72–1.07)	0.88 (0.72–1.07)	0.91 (0.75–1.11)
Promotion	0.87 (0.71–1.07)	0.88 (0.72–1.09)	0.89 (0.72–1.10)	0.90 (0.73–1.12)
Over-commitment	1.20 (0.95–1.50)	1.20 (0.95–1.51)	1.17 (0.92–1.48)	1.16 (0.90–1.48)
Pseudo *R*^2^	0.08	0.09	0.10	0.11
AIC	735.7711	730.9747	739.523	741.8441

Bold indicates statistical significance

*AIC* Akaike Information Criterion, *95%CI* 95% confidence interval

*p < 0.5, **p < 0.01

^a^Model 1: Adjusted for MDD status in the beginning of the follow-up

^b^Model 2: model 1 additionally adjusted for age, sex, and follow-up duration

^c^Model 3: model 2 additionally adjusted for physical activity, smoking status, anxiety disorders, alcohol use disorder, and illicit drug use disorder in the beginning of the follow-up

^d^Model 4: model 3 additionally adjusted for burnout dimensions (exhaustion, cynicism, and professional efficacy) measured in the beginning of the follow-up

**Table 3:** Results of logistic regression models of the associations of work stress modeled as Effort-Reward Imbalance (ERI), or its dimensions, and over-commitment in the beginning of the follow-up with the incidence of Major Depressive Disorder during the follow-up in the sub-sample with no lifetime history of MDD in the beginning of the follow-up (*n* = 485)

Predictors	Model^a^ Odds ratio (95%CI)	Model^b^ Odds ratio (95%CI)	Model^c^ Odds ratio (95%CI)	Model^d^ Odds ratio (95%CI)
Effort-reward ratio	1.51* (1.04, 2.18)	1.55* (1.06–2.27)	1.58* (1.06–2.36)	1.31 (0.87–1.95)
Over-commitment	1.03 (0.69, 1.53)	1.02 (0.68, 1.53)	0.98 (0.64, 1.50)	0.90 (0.58, 1.42)
Pseudo *R*^2^	0.04	0.05	0.10	0.12
AIC	252.9744	256.7569	260.5829	262.4568
Effort	1.30 (0.88, 1.91)	1.34 (0.90–1.99)	1.40 (0.92–2.13)	1.19 (0.76–1.86)
Security	1.04 (0.73, 1.49)	1.02 (0.71–1.46)	1.03 (0.70–1.52)	1.14 (0.75–1.73)
Esteem	0.65* (0.47, 0.92)	0.66* (0.47–0.93)	0.62** (0.43–0.88)	0.66* (0.45–0.95)
Promotion	0.93 (0.65, 1.34)	0.93 (0.64–1.34)	0.99 (0.66–1.46)	1.04 (0.68–1.57)
Over-commitment	1.05 (0.70, 1.57)	1.02 (0.68–1.55)	0.97 (0.62–1.50)	0.91 (0.57–1.45)
Pseudo *R*^2^	0.05	0.05	0.11	0.13
AIC	256.4946	260.8344	263.7926	265.2351

Bold indicates statistical significance

*AIC* Akaike Information Criterion, *95%CI* 95% confidence interval

*p < 0.05, **p < 0.01

^a^Model 1: unadjusted

^b^Model 2: model 1 additionally adjusted for age, sex, and follow-up duration

^c^Model 3: model 2 additionally adjusted for physical activity, smoking status, anxiety disorders, alcohol use disorder, and illicit drug use disorder in the beginning of the follow-up

^d^Model 4: model 3 additionally adjusted for burnout dimensions (exhaustion, cynicism, and professional efficacy) measured in the beginning of the follow-up

**Table 4:** Results of logistic regression models of the associations of work stress modeled as Effort-Reward Imbalance (ERI), or its dimensions, and over-commitment in the beginning of the follow-up with the occurrence of a new major depressive episode during the follow-up period in the sub-sample of participants with a history of remitted major depressive disorder in the beginning of the follow-up (*n* = 474)

Predictors	Model^a^ Odds ratio (95%CI)	Model^b^ Odds ratio (95%CI)	Model^c^ Odds ratio (95%CI)	Model^d^ Odds ratio (95%CI)
Effort-reward ratio	1.24 (0.99, 1.57)	1.23 (0.97–1.56)	1.22 (0.97–1.55)	1.14 (0.87–1.49)
Over-commitment	1.23 (0.95, 1.59)	1.24 (0.96, 1.61)	1.24 (0.95, 1.62)	1.25 (0.94, 1.66)
Pseudo *R*^2^	0.02	0.05	0.06	0.07
AIC	483.0358	477.3112	489.4173	493.5337
Effort	1.05 (0.78, 1.38)	1.04 (0.78–1.38)	1.04 (0.78–1.39)	1.004 (0.73–1.37)
Security	0.82 (0.65, 1.02)	0.80 (0.64–1.01)	0.82 (0.65–1.03)	0.84 (0.66, 1.06)
Esteem	0.96 (0.66, 1.09)	0.98 (0.77–1.26)	0.99 (0.77–1.28)	1.03 (0.80–1.33)
Promotion	0.85 (0.66, 1.09)	0.87 (0.67–1.13)	0.87 (0.66–1.23)	0.87 (0.66–1.14)
Over-commitment	1.25 (0.95, 1.66)	1.26 (0.95–1.67)	1.26 (0.94–1.68)	1.27 (0.94–1.72)
Pseudo *R*^2^	0.03	0.06	0.06	0.06
AIC	484.249	478.9636	491.5369	496.1128

*AIC* Akaike Information Criterion, *95%CI* 95% confidence interval

*p < 0.05, **p < 0.01

^a^Model 1: unadjusted

^b^Model 2: model 1 additionally adjusted for age, sex, and follow-up duration

^c^Model 3: model 2 additionally adjusted for physical activity, smoking status, anxiety disorders, alcohol use disorder, and illicit drug use disorder in the beginning of the follow-up

^d^Model 4: model 3 additionally adjusted for burnout dimensions (exhaustion, cynicism, and professional efficacy) measured in the beginning of the follow-up

### Interactions between ERI ratio (imbalance) and burnout dimensions regarding MDD during a four-year follow-up

Models testing the effect of burnout dimensions at the first assessment on the prospective associations between ERI and MDE onset during a four-year follow-up did not reveal any significant interaction between ERI ratio at the first assessment and burnout dimensions [*p*-value of interaction test: exhaustion (*p* = 0.74), cynicism (*p* = 0.85), or professional efficacy (*p* = 0.56)]. Similar results were found among participants never depressed before the first assessment [*p*-value of interaction test: exhaustion (*p* = 0.35), cynicism (*p* = 0.23), or professional efficacy (*p* = 0.77)] and participants with remitted MDD [*p*-value of interaction test: exhaustion (*p* = 0.70), cynicism (*p* = 0.93), or professional efficacy (*p* = 0.32)].

## Discussion

### Main findings

The main results of this study highlight the longitudinal association between exposure to work-related stress modeled as effort-reward imbalance and MDE onset in participants with no current MDE at the first assessment. However, it seems that burnout had a role in the association between the effort-reward imbalance and incident MDD. This may be explained by the cross-sectional correlation between exposure to work-related stress and burnout, either burnout and exposure to work-related stress may occur simultaneously, or burnout could potentially increase job stress, or vice versa. Future research is needed to answer the question about the temporality of the association between burnout and exposure to work-related stress with shorter length of follow-up and across multiple assessments. This finding concurs with the current understanding of burnout in the International Classification of Diseases, the eleventh revision (ICD-11) as a syndrome resulting from chronic workplace stress that has not been successfully managed and a risk state (but not a disease itself) that may lead to physical and mental diseases (here MDD) [70]. Future research should thus consider burnout, particularly when assessing the association between the effort-reward ratio and the incidence of MDD. None of the ERI dimensions nor over-commitment were associated with an increased onset of MDE after the four-year follow-up. However, for participants who were never depressed until the first assessment, only the esteem dimension was associated with a decreased incidence of MDD after the four-year follow-up, suggesting that higher esteem protects workers from developing MDD.

### The role of burnout in the association between exposure to work-related stress and depression

Two reviews concluded that the effort-reward ratio increased the risk of depression [52, 59]. We found similar results in this study although the effect size slightly decreased when adjusting for the three burnout dimensions. However, the original studies included in the two reviews did not consider the role of burnout in the association between effort-reward ratio and depression. Additionally, some of the original studies included in those reviews used self-report assessments for depression [5, 11, 14, 33, 36, 43, 69, 72]. Nonetheless, the assessment of the role of burnout in the association between effort-reward imbalance and MDD seemed essential. Burnout could act either as a mediator, an effect modifier, or a confounding factor in the association between effort-reward imbalance and MDD. However, exposure to work-related stress was not longitudinally associated with an increase in the scores of burnout dimensions after four years of follow-up in the CoLaus|PsyCoLaus cohort [56] therefore burnout is unlikely a mediator in this cohort. Additionally, the results of interaction tests between effort-reward imbalance and burnout dimensions at the first assessment indicated that burnout is not an effect modifier for the association between effort-reward imbalance and MDD. Finally, after adjustment for burnout, the association between exposure to work-related stress and MDD remained significant with a slight decrease in effect size. However, the association between effort-reward imbalance and the incidence of MDD was not statistically significant in the subsample of participants who never had been depressed until the first assessment. This could be explained by the cross-sectional correlation between exposure to work-related stress and burnout. Therefore, focusing on reducing the effort-reward imbalance ratio (not necessarily overcommitment) to reduce burnout or vice versa might help reduce the risk of the first MDD episode among workers. It is worth noting that the effortrReward imbalance represents the extrinsic component of work-related stress, which makes interventions at the organizational level a target for change, as opposed to over-commitment, which denotes the intrinsic component of work-related stress [65]. However, the effect sizes of the associations between over-commitment and MDD were still higher compared to the effect sizes of the associations between other ERI dimensions and MDD. This may be explained by the fact that the effect of over-commitment could be attenuated by the other ERI dimensions.

It is noteworthy to mention that the first analysis (Aim 1) included participants with remitted MDD and participants who were exempt from lifetime MDD in the beginning of the follow-up. Although participants with remitted MDD did not have a current MDE in the beginning of the follow-up and, therefore, were included in this analysis, they may have had residual symptoms, which might increase the risk of another MDE in these participants. However, to address this issue, we presented two separate analyses for participants free from lifetime MDD (Aim 2) and participants with remitted MDD (Aim 3).

### Exposure to work-related stress dimensions and depression

Our results indicated that there are no associations between ERI dimensions and MDD diagnosis, except for the esteem dimension in the analysis of the subsample of never-depressed participants until the first assessment. The esteem dimension of ERI represents recognition (socioemotional reward) of one’s work performance [58]. We, therefore, suggest that future interventional studies focus on esteem since it can help protect workers from developing the first episode of MDD. Eight out of 14 longitudinal studies did not report the association between the ERI dimensions and depression, and only reported the association for the effort-reward imbalance [26, 33, 36, 42, 43, 51, 60, 68]. The results of one out of six studies were in line with our findings and concluded that the dimensions of effort and reward were not associated with depression [1]. Contrary to our findings, the results of five studies showed significant associations between the dimensions of effort and reward and depression [5, 14, 23, 69, 72]. However, depression was measured using self-reported assessment tools in four of these studies [5, 14, 69, 72]. The outcome of the fifth study was disability pension due to depression [23]. The self-reported assessment tools or different outcome definitions of depression may explain the differences between the results of the five studies and our findings. Structured or semi-structured diagnostic interviews are the “gold standard” for confirming the diagnoses of psychiatric disorders [13]. Therefore, using the diagnosis of MDD when available is clearly preferable to the use of self-reported depression.

Comparably few studies assessed the association between over-commitment and depression [5, 14, 33, 69]. Three studies reported a significant association between over-commitment and depression, two studies used a self-report assessment tool for depression, whereas the third study used the quantiles of over-commitment as a predictor instead of the continuous score [14]. Nonetheless, one study reported that over-commitment was not associated with depression [5], similar to the findings of our study.

### Strengths and limitations

This study has several strengths: the longitudinal study design to examine the association between ERI, over-commitment, and MDD with a relatively long follow-up the objective assessment of the outcome (i.e., MDD) using diagnostic interviews, the control for a set of potential covariates and burnout, and objective assessment of some of the confounders by study clinicians (e.g., anxiety disorders, alcohol use disorder, and illicit drug use disorder). The main limitation of this study is the self-reported measurement of the predictors (i.e., ERI, and over-commitment) and burnout which may lead to common method variance. A more objective assessment of burnout is thus recommended in future studies, for example by using a hetero-evaluation tool [10] that can be filled by healthcare professionals and was validated in Belgium and Switzerland [32, 39] or standardized guidelines. For example, in the Netherlands, there are diagnostic criteria for burnout that rely on the work-related neurasthenia in the ICD-10 [54] and in Switzerland, the Swiss Network of Burnout Experts published practical recommendations to deal with burnout in 2016 [21, 22]. It is worth mentioning that the Likert scale answer format has been revised to a one-step procedure with four categories (1) strongly disagree, (2) disagree, (3) agree, and (4) strongly agree), based on recommendations by Tsutsumi et al. [64] and Siegrist et al. [61]. While no significant differences in psychometric analyses were found between the two procedures, the one-step approach resulted in significantly higher response rates. Therefore, it is recommended that future studies adopt this method.

## Implication of the findings

The imbalance between effort and reward led to MDD longitudinally in the CoLaus|PsyCoLaus population-based cohort. Our findings suggest that burnout plays a role in the longitudinal association between exposure to work-related stress and MDD in CoLaus|PsyCoLaus. Future research should explore whether burnout and exposure to work-related stress occur simultaneously, or one proceeds the other. Additionally, it may be worthwhile to investigate the role of burnout as a mediator for the association between exposure to work-related stress and MDD in other population-based cohorts where exposure to work-related stress leads to burnout longitudinally and burnout and ERI are measured across three different follow-ups at least. Since the second assessment was conducted partially during the COVID-19 pandemic (2018–2020), it is important to consider the potential effects of the pandemic on incident MDE and response rates in the second assessment of this study. The pandemic has been shown to exacerbate exposure to adverse psychosocial conditions and increase the risk of depressive disorders [55]. Therefore, it is essential to bear in mind this significant mental health issue when interpreting the results of this study.

## Conclusions

There is a longitudinal association between effort-reward imbalance and the onset of MDE in the CoLaus|PsyCoLaus cohort even after adjustment for the burnout dimensions. None of the ERI dimensions nor over-commitment were associated with the onset of MDE. However, it seems that burnout may play a role in the association between effort-reward imbalance and the MDD in never-depressed participants (i.e., incidence MDD). Focusing on reducing ERI (not necessarily overcommitment) to reduce burnout or vice versa might help reduce the risk of incident MDD. The results of this study suggest future research to consider the potential role of burnout when assessing the association between exposure to work-related stress and MDD. Such research could assess whether burnout prevention can reduce the risk of a first episode of MDD in previously non-depressed workers with job stress.

## Supplementary Information

Below is the link to the electronic supplementary material.

Supplementary file1 (DOCX 49 KB)

## Data Availability

The data of CoLaus|PsyCoLaus study used in this article cannot be fully shared as they contain potentially sensitive personal information on participants. According to the Ethics Committee for Research of the Canton of Vaud, sharing these data would be a violation of the Swiss legislation with respect to privacy protection. However, coded individual-level data that do not allow researchers to identify participants are available upon request to researchers who meet the criteria for data sharing of the CoLaus|PsyCoLaus Datacenter (CHUV, Lausanne, Switzerland). Any researcher affiliated to a public or private research institution who complies with the CoLaus|PsyCoLaus standards can submit a research application to research.colaus@chuv.ch or research.psycolaus@chuv.ch. Proposals requiring baseline data only, will be evaluated by the baseline (local) Scientific Committee (SC) of the CoLaus and PsyCoLaus studies. Proposals requiring follow-up data will be evaluated by the follow-up (multicentric) SC of the CoLaus|PsyCoLaus cohort study. Detailed instructions for gaining access to the CoLaus|PsyCoLaus data used in this study are available at www.colauspsycolaus.ch/professionals/how-to-collaborate/.
