# Supplementary material for: Longitudinal association of exposure to work-related stress with major depressive disorder and the role of occupational burnout in this association in the general population
Source: Soc Psychiatry Psychiatr Epidemiol. 2024 Aug 31;60(3):593–606. doi: 10.1007/s00127-024-02735-w (PMC11870932; doi:10.1007/s00127-024-02735-w)
Supplement: Supplementary file 1 — Supplementary file1 (DOCX 49 KB) [file 127_2024_2735_MOESM1_ESM.docx]

**Supplementary Table S1. Correlation matrix for the independent variables among participants without current major depressive episode at the first assessment, n=959**

|  | Age | Effort-Reward ratio | Effort | Security | Esteem | Promotion | Over-commitment | Exhaustion | Cynicism | Professional efficacy | Length of follow-up |
| --- | --- | --- | --- | --- | --- | --- | --- | --- | --- | --- | --- |
| Age | 1 |  |  |  |  |  |  |  |  |  |  |
| Effort-Reward ratio | -0.12 | 1 |  |  |  |  |  |  |  |  |  |
| Effort | -0.14 | 0.79 | 1 |  |  |  |  |  |  |  |  |
| Security | 0.09 | -0.58 | -0.35 | 1 |  |  |  |  |  |  |  |
| Esteem | 0.07 | -0.36 | -0.03 | 0.22 | 1 |  |  |  |  |  |  |
| Promotion | -0.01 | -0.36 | 0.05 | 0.14 | 0.45 | 1 |  |  |  |  |  |
| Over-commitment | 0.02 | 0.49 | 0.58 | -0.29 | -0.04 | -0.05 | 1 |  |  |  |  |
| Exhaustion | -0.08 | 0.50 | 0.51 | -0.32 | -0.16 | -0.10 | 0.48 | 1 |  |  |  |
| Cynicism | -0.06 | 0.46 | 0.40 | -0.35 | -0.22 | -0.19 | 0.33 | 0.61 | 1 |  |  |
| Professional efficacy | 0.03 | -0.11 | -0.03 | 0.11 | 0.07 | 0.11 | -0.02 | -0.13 | -0.16 | 1 |  |
| Length of follow-up | -0.08 | -0.05 | -0.05 | 0.03 | 0.05 | 0.0006 | -0.06 | -0.03 | -0.05 | 0.07 | 1 |

**Supplementary Table S2. Correlation matrix for the independent variables in the sub-sample of participants never depressed until the first assessment, n=485**

|  | Age | Effort-Reward ratio | Effort | Security | Esteem | Promotion | Over-commitment | Exhaustion | Cynicism | Professional efficacy | Length of follow-up |
| --- | --- | --- | --- | --- | --- | --- | --- | --- | --- | --- | --- |
| Age | 1 |  |  |  |  |  |  |  |  |  |  |
| Effort-Reward ratio | -0.10 | 1 |  |  |  |  |  |  |  |  |  |
| Effort | -0.16 | 0.79 | 1 |  |  |  |  |  |  |  |  |
| Security | 0.1 | -0.56 | -0.34 | 1 |  |  |  |  |  |  |  |
| Esteem | -0.07 | -0.28 | 0.11 | 0.14 | 1 |  |  |  |  |  |  |
| Promotion | 0.05 | -0.34 | -0.04 | 0.21 | 0.39 | 1 |  |  |  |  |  |
| Over-commitment | 0.05 | 0.47 | 0.55 | -0.30 | -0.05 | -0.06 | 1 |  |  |  |  |
| Exhaustion | -0.04 | 0.52 | 0.50 | -0.35 | -0.12 | -0.18 | 0.48 | 1 |  |  |  |
| Cynicism | -0.003 | -0.09 | -0.01 | 0.12 | 0.05 | 0.06 | 0.03 | -0.12 | 1 |  |  |
| Professional efficacy | -0.02 | 0.38 | 0.32 | -0.33 | -0.15 | -0.21 | 0.31 | 0.56 | -0.09 | 1 |  |
| Length of follow-up | -0.08 | -0.09 | -0.11 | 0.07 | -0.04 | 0.03 | -0.1 | -0.08 | 0.09 | -0.09 | 1 |

**Supplementary Table S3. Correlation matrix for the independent variables in the sub-sample of participants with remitted major depressive disorder at the first assessment, n=474**

|  | Age | Effort-Reward ratio | Effort | Security | Esteem | Promotion | Over-commitment | Exhaustion | Cynicism | Professional efficacy | Length of follow-up |
| --- | --- | --- | --- | --- | --- | --- | --- | --- | --- | --- | --- |
| Age | 1 |  |  |  |  |  |  |  |  |  |  |
| Effort-Reward ratio | -0.12 | 1 |  |  |  |  |  |  |  |  |  |
| Effort | -0.10 | 0.78 | 1 |  |  |  |  |  |  |  |  |
| Security | 0.07 | -0.60 | -0.35 | 1 |  |  |  |  |  |  |  |
| Esteem | 0.05 | -0.41 | 0.02 | 0.14 | 1 |  |  |  |  |  |  |
| Promotion | 0.07 | -0.36 | -0.01 | 0.22 | 0.49 | 1 |  |  |  |  |  |
| Over-commitment | 0.02 | 0.50 | 0.60 | -0.28 | -0.04 | -0.02 | 1 |  |  |  |  |
| Exhaustion | -0.08 | 0.47 | 0.51 | -0.30 | -0.08 | -0.13 | 0.48 | 1 |  |  |  |
| Cynicism | -0.08 | 0.50 | 0.44 | -0.36 | -0.22 | -0.22 | 0.33 | 0.64 | 1 |  |  |
| Professional efficacy | 0.05 | -0.12 | -0.04 | 0.11 | 0.17 | 0.08 | -0.07 | -0.13 | -0.24 | 1 |  |
| Length of follow-up | -0.09 | 0.003 | 0.02 | -0.02 | 0.03 | 0.07 | -0.01 | 0.04 | 0.001 | 0.04 | 1 |

**Supplementary Table S4. Descriptive statistics of the study variables among participants without current major depressive episode at the first assessment and with remitted MDD according to major depressive disorder status during the four-year follow-up, n=959**

|  | **MDD during the four-year follow-up** | |  | | |
| --- | --- | --- | --- | --- | --- |
|  | **No MDD** | **MDD** | **Statistic** | **p-value** |  |
| ***Number of participants*** | *823* | *136* |  | |  |
| **Demographic characteristics** |  |  |  |  |  |
| Age at the first assessment (years), median (IQR) | 56.1 (9.2) | 55.45 (7.6) | z=1.15 | 0.25 |  |
| Male sex, % | 52.1 | 32.4 | χ2=18.26 | **<0.001** |  |
| Length of follow-up (years), mean (SD) | 3.61 (0.54) | 3.67 (0.54) | t=-1.21 | 0.23 |  |
| **Behavioral characteristics at the first assessment** |  |  |  |  |  |
| Smoking status, % |  |  | χ2=1.16 | 0.56 |  |
| Active smokers | 9.5 | 8.8 |  |  |  |
| Former smokers | 48.7 | 53.7 |  |  |  |
| Non-smokers | 41.8 | 37.5 |  |  |  |
| Physical activity^a^, % | 80.0 | 79.4 | χ2=0.02 | 0.88 |  |
| **Lifetime psychiatric disorder until the first assessment** |  |  |  |  |  |
| Illicit drug use disorder (abuse/dependence), % |  |  | χ2=0.91 | 0.64 |  |
| Current | 0.2 | 0.7 |  |  |  |
| Remitted | 8.1 | 8.1 |  |  |  |
| Never | 91.6 | 91.2 |  |  |  |
| Alcohol use disorder (abuse/dependence), % |  |  | χ2=1.88 | 0.39 |  |
| Current | 1.9 | 3.7 |  |  |  |
| Remitted | 11.2 | 9.6 |  |  |  |
| Never | 86.9 | 86.8 |  |  |  |
| Anxiety disorders^b^, % |  |  | χ2=17.85 | **<0.001** |  |
| Current | 1.7 | 4.4 |  |  |  |
| Remitted | 17.9 | 30.9 |  |  |  |
| Never | 80.4 | 64.7 |  |  |  |
| **Burnout^c^ at the first assessment, median (IQR)** |  |  |  |  |  |
| Exhaustion | 1 (1.4) | 1.5 (1.7) | z=-5.08 | **<0.001** |  |
| Cynicism | 1.4 (1.6) | 1.6 (2.2) | z=-3.69 | **<0.001** |  |
| Professional efficacy | 4.33 (1.17) | 4.66 (1.16) | z\|=1.82 | 0.07 |  |
| **Effort-Reward Imbalance at the first assessment,** |  |  |  |  |  |
| Effort-Reward ratio, median (IQR) | 0.23 (0.11) | 0.27(0.15) | z=-4.38 | **<0.001** |  |
| Effort, median (IQR) | 11 (5) | 12.60 (6) | z=-3.11 | **0.002** |  |
| Reward |  |  |  |  |  |
| Security, median (IQR) | 8 (2) | 8 (2) | z=3.28 | **<0.001** |  |
| Esteem, mean (SD) | 11.71 (1.83) | 11.15 (2.74) | t=3.07 | **<0.01** |  |
| Promotion, mean (SD) | 8.55 (1.65) | 8.05 (1.78) | t=3.23 | **<0.001** |  |
| Over-commitment | 13.65 (3.57) | 14.87 (3.90) | t=-3.64 | **<0.001** |  |

Descriptive statistics are presented as mean (standard deviation) or median (interquartile range) for continuous variables and percentage for categorical variables. Pearson’s chi-square test (χ^2^) for categorical variables and Student test (t) or Wilcoxon signed-rank test (z) for continuous variables were used as appropriate.

MDD=major depressive disorder; SD=standard deviation; IQR: Interquartile range

^a^ at least once a week

^b^ generalized anxiety disorder, panic disorder, agoraphobia, social phobia

^c^ measured using Maslach Burnout Inventory (MBI)

**Supplementary Table S5. Descriptive statistics of the study variables in the sub-sample of participants never depressed until the first assessment according to major depressive disorder status during the four-year follow-up, n=485**

|  | **MDD diagnosis during the four-year follow-up** | |  | | |
| --- | --- | --- | --- | --- | --- |
|  | **No MDD** | **MDD** | **Statistic** | **p-value** |  |
| ***Number of participants*** | *449* | *36* |  | |  |
| **Demographic characteristics** |  |  |  |  |  |
| Age at the first assessment (years), median (IQR) | 56.4 (9.2) | 57.35 (7.6) | z=0.56 | 0.59 |  |
| Male sex, % | 62.6 | 55.6 | χ2=0.70 | 0.40 |  |
| Length of follow-up (years), mean (SD) | 3.64 (0.54) | 3.67 (0.54) | t=-0.28 | 0.78 |  |
| **Behavioral characteristics at the first assessment** |  |  |  |  |  |
| Smoking status, % |  |  | χ2=3.66 | 0.16 |  |
| Active smokers | 10 | 8.3 |  |  |  |
| Former smokers | 44.8 | 61.1 |  |  |  |
| Non-smokers | 45.2 | 30.6 |  |  |  |
| Physical activity ^a^, % | 81.3 | 77.8 | χ2=0.27 | 0.61 |  |
| **Lifetime psychiatric disorder until the first assessment** |  |  |  |  |  |
| Illicit drug use disorder (abuse/dependence), % |  |  | χ2=5.47 | 0.07 |  |
| Current | 0.2 | 2.8 |  |  |  |
| Remitted | 6.7 | 8.3 |  |  |  |
| Never | 93.1 | 88.9 |  |  |  |
| Alcohol use disorder (abuse/dependence), % |  |  | χ2=0.99 | 0.61 |  |
| Current | 2.7 | 5.6 |  |  |  |
| Remitted | 10.9 | 11.1 |  |  |  |
| Never | 86.4 | 83.3 |  |  |  |
| Anxiety disorders ^b^, % |  |  | χ2=12.35 | **<0.01** |  |
| Current | 0.9 | 5.6 |  |  |  |
| Remitted | 13.1 | 27.8 |  |  |  |
| Never | 86 | 66.7 |  |  |  |
| **Burnout**^c^ **at the first assessment, median (IQR)** |  |  |  |  |  |
| Exhaustion | 0.8 (1.4) | 1.4 (1.3) | z=-3.74 | **<0.001** |  |
| Cynicism | 1.2 (1.8) | 1.5 (2.1) | z=-1.98 | **0.05** |  |
| Professional efficacy | 4.83 (0.83) | 4.58 (0.92) | z=1.78 | 0.07 |  |
| **Effort-Reward Imbalance at the first assessment** |  |  |  |  |  |
| Effort-Reward ratio, median (IQR) | 0.22 (0.11) | 0.24 (0.15) | z=-2.23 | **0.03** |  |
| Effort, median (IQR) | 11 (5) | 12 (5.5) | z=-1.58 | 0.11 |  |
| Reward |  |  |  |  |  |
| Security, median (IQR) | 8 (1) | 8 (2) | z=0.96 | 0.34 |  |
| Esteem, mean (SD) | 11.77 (1.57) | 10.86 (2.54) | t=3.18 | **<0.01** |  |
| Promotion, mean (SD) | 8.63 (1.52) | 8.14 (1.96) | t=1.81 | 0.07 |  |
| Over-commitment, mean (SD) | 13.50 (3.50) | 14.44 (3.94) | t=-1.55 | 0.12 |  |

Descriptive statistics are presented as mean (standard deviation) or median (interquartile range) for continuous variables and percentage for categorical variables. Pearson’s chi-square test (χ^2^) for categorical variables and Student test (t) or Wilcoxon signed-rank test (z)_for continuous variables were used as appropriate.

MDD=major depressive disorder; SD=standard deviation; IQR: Interquartile range

^a^ at least once a week

^b^ generalized anxiety disorder, panic disorder, agoraphobia, social phobia

^c^ measured using Maslach Burnout Inventory (MBI)

**Supplementary Table S6. Descriptive statistics of the study variables in the sub-sample of participants without a current Major Depressive Episode (MDE) and with remitted Major Depressive Disorder (MDD) at the first assessment according to the MDD status during the four-year follow-up, n=474**

|  | **MDD diagnosis during the four-year follow-up** | |  | | |
| --- | --- | --- | --- | --- | --- |
|  | **No MDD** | **MDD** | **Statistic** | **p-value** |  |
| ***Number of participants*** | *374* | *100* |  | |  |
| **Demographic characteristics** |  |  |  |  |  |
| Age at the first assessment (years), median (IQR) | 55.3 (8.9) | 55.1 (7.4) | z=1.13 | 0.26 |  |
| Male sex, % | 39.6 | 24 | χ2=8.28 | **0.004** |  |
| Length of follow-up (years), mean (SD) | 3.57 (0.54) | 3.67 (0.54) | t=-1.65 | 0.10 |  |
| **Behavioral characteristics at the first assessment** |  |  |  |  |  |
| Smoking status, % |  |  | χ2=0.20 | 0.90 |  |
| Active smokers | 8.8 | 9 |  |  |  |
| Former smokers | 53.5 | 51 |  |  |  |
| Non-smokers | 37.7 | 40 |  |  |  |
| Physical activity ^a^, % | 78.3 | 80 | χ2=0.13 | 0.72 |  |
| **Lifetime psychiatric disorder until the first assessment** |  |  |  |  |  |
| Illicit drug use disorder (abuse/dependence), % |  |  | χ2=0.61 | 0.74 |  |
| Current | 0.3 | 0 |  |  |  |
| Remitted | 9.9 | 8 |  |  |  |
| Never | 89.8 | 92 |  |  |  |
| Alcohol use disorder (abuse/dependence), % |  |  | χ2=2.44 | 0.30 |  |
| Current | 1.1 | 3 |  |  |  |
| Remitted | 11.5 | 9 |  |  |  |
| Never | 87.4 | 88 |  |  |  |
| Anxiety disorders ^b^, % |  |  | χ2=3.76 | 0.15 |  |
| Current | 2.7 | 4 |  |  |  |
| Remitted | 23.5 | 32 |  |  |  |
| Never | 73.8 | 64 |  |  |  |
| **Burnout**^c^ **at the first assessment, median (IQR)** |  |  |  |  |  |
| Exhaustion | 1.4 (1.6) | 1.6 (1.9) | z-2.13 | **0.03** |  |
| Cynicism | 1.6 (1.6) | 1.8 (2.3) | z=-2.30 | **0.02** |  |
| Professional efficacy | 4.67 (1) | 4.67 (1.25) | z=0.68 | 0.50 |  |
| **Effort-Reward Imbalance at the first assessment** |  |  |  |  |  |
| Effort-Reward ratio, median (IQR) | 0.23 (0.12) | 0.27 (0.16) | z=-2.91 | **0.004** |  |
| Effort, median (IQR) | 13 (6) | 12 (6) | z=-1.90 | 0.06 |  |
| Reward |  |  |  |  |  |
| Security, median (IQR) | 8 (2) | 8 (3) | z=2.97 | **0.003** |  |
| Esteem, mean (SD) | 11.63 (2.1) | 11.25 (2.81) | t=1.51 | 0.13 |  |
| Promotion, mean (SD) | 8.46 (1.79) | 8.02 (1.71) | t=2.20 | **0.03** |  |
| Over-commitment, mean (SD) | 13.84 (3.63) | 15.02 (3.89) | t=-2.85 | **<0.01** |  |

Descriptive statistics are presented as mean (standard deviation) or median (interquartile range) for continuous variables and percentage for categorical variables. Pearson’s chi-square test (χ^2^) for categorical variables and Student test (t) or Wilcoxon signed-rank test (z)_for continuous variables were used as appropriate.

MDD=major depressive disorder; SD=standard deviation; IQR: Interquartile range

^a^ at least once a week

^b^ generalized anxiety disorder, panic disorder, agoraphobia, social phobia

^c^ measured using Maslach Burnout Inventory (MBI)
